# Association of p-glycoprotein and bile salt export pump gene polymorphisms with advanced liver disease in hepatitis C virus infected patients

**DOI:** 10.1590/0074-02760250173

**Published:** 2026-07-20

**Authors:** Letícia Bomfim Campos, Nathália Alves Araújo de Almeida, Marcia Maria Amendola Pires, Carlos Eduardo Brandão-Mello, Livia Melo Villar, José Júnior França de Barros, Vanessa Salete de Paula

**Affiliations:** 1 Fundação Oswaldo Cruz-Fiocruz, Instituto Oswaldo Cruz, Laboratório de Virologia e Parasitologia Molecular, Rio de Janeiro, RJ, Brasil; 2 Fundação Oswaldo Cruz-Fiocruz, Instituto Nacional de Infectologia Evandro Chagas, Laboratório de Pesquisa Clínica em Neuroinfecções, Rio de Janeiro, RJ, Brasil; 3 Universidade Federal do Estado do Rio de Janeiro, Hospital Universitário Gaffrée e Guinle, Serviço de Gastroenterologia, Rio de Janeiro, RJ, Brasil; 4 Fundação Oswaldo Cruz-Fiocruz, Instituto Oswaldo Cruz, Laboratório de Hepatites Virais, Rio de Janeiro, RJ, Brasil

**Keywords:** hepatitis C, ATP-binding cassette, ABCB1, ABCB11, drug resistance, genetic factors

## Abstract

**BACKGROUND:**

Single-nucleotide polymorphisms (SNPs) can influence the hepatitis C virus (HCV) infection and progression. *ABCB1*-gene SNPs - c.1236C>T, c.2677G>T and c.3435C>T - are associated with drug efficacy, hepatotoxicity, and liver injury. *ABCB11* c.1331T>C is associated with cholestasis and altered bilirubin levels, potentially worsening liver disease.

**OBJECTIVE:**

To investigate the impact of *ABCB1* and *ABCB11* SNPs on disease progression in chronic HCV patients.

**METHODS:**

A total of 232 HCV patients unresponsive to conventional therapy were analysed. Serum samples were genotyped by quantitative polymerase chain reaction (qPCR), and the genotype and allele-based analysis was performed using RStudio.

**FINDINGS:**

Cirrhosis was present in 59.1% of patients, along with diabetes (28.4%) and hepatic steatosis (46.1%). The most frequent *ABCB1* variant allele was c.3435C>T (36.8%), followed by c.1236C>T (30.8%) and c.2677G>T (26.7%). The *ABCB11* c.1331CC genotype was observed in 31.3% of the cohort. Genotypes 1236TT and 2677TT and their alleles were associated with lower total cholesterol. 2677TT genotype and 2677T allele were associated with lower high-density lipoprotein. Patients with 1331CC genotype had higher aspartate aminotransferase levels, and the 1331CC genotype was a risk factor for cirrhosis. A fully variant combined allele (1236T and 2677T and 3435T and 1331C) was associated with higher alpha-fetoprotein and lower cholesterol.

**MAIN CONCLUSIONS:**

*ABCB1* and *ABCB11* SNPs are associated with worse clinical outcomes in HCV, underscoring their relevance in disease monitoring.

Hepatitis C virus (HCV) is the leading cause of chronic hepatitis, with 50 million individuals estimated to be chronically infected with the virus worldwide and 1 million new infections occurring per year.^1^ HCV-infected individuals are at high risk of developing liver disease that can progress to cirrhosis and hepatocellular carcinoma (HCC), which is the most common primary liver cancer and the main reason for liver transplantation.^2^ ^,^ ^3^ During hepatocyte infection, biochemical alterations may occur, including increased serum levels of hepatic and canalicular membrane enzymes, such as aspartate aminotransferase (AST), alanine aminotransferase (ALT), and gamma-glutamyl transferase (GGT), which are associated with cholestasis and HCV-induced liver damage.^4^ Given that the liver plays an important role in lipid metabolism, the lipid profile may deteriorate in patients with more severe disease, including uncompensated cirrhosis, with reduced levels of cholesterol and high-density lipoprotein (HDL) ensuing.^5^ ^,^ ^6^ ^,^ ^7^

Several viral and host factors are associated with increased susceptibility to HCV infection and liver disease progression. These include age, male sex, obesity, insulin resistance, type 2 diabetes, co-infection with hepatitis B virus or human immunodeficiency virus (HIV), hepatic steatosis,^8^ ^,^ ^9^ and vitamin D deficiency.^10^ ^,^ ^11^ ^,^ ^12^ ^,^ ^13^

ATP-binding cassette (ABC) transporters, which are present in several species, are transmembrane proteins that harvest energy from ATP hydrolysis to transport a variety of molecules, including drugs.^14^ One of the most-studied proteins in this family is P-glycoprotein, which functions as a drug efflux pump. P-glycoprotein is encoded by the *ABCB1*/multidrug resistance 1 (*MDR1*) gene, the single nucleotide polymorphisms (SNPs) of which have been associated with hepatotoxicity and resistance to multiple drugs (usually antineoplastic).^15^ ^,^ ^16^ ^,^ ^17^

Three polymorphisms in the *ABCB1* gene (c.3435C>T, c.2677G>T, and c.1236C>T) are associated with differences in its expression and in the activity of its translated protein. The SNP c.3435C>T (rs1045642), located in exon 26 of *ABCB1*, is a synonymous polymorphism (p.Ile1145Ile) that is associated with reduced protein expression.^18^ The c.2677G>T (rs2032582) variant in exon 21 is a non-synonymous polymorphism (p.Ala893Ser), resulting in an amino acid substitution located at the cytoplasmic region within the second transmembrane domain of the *ABCB1* protein, potentially affecting its activity and substrate specificity.^18^ The c.1236C>T (rs1128503) genetic variant in exon 12, a synonymous polymorphism (p.Gly412Gly), is associated with changes in protein expression.^18^

The *ABCB11* gene encodes the bile salt export pump (BSEP), another important protein in the *ABC* transporter family that is found mainly in the apical (canalicular) membrane of hepatocytes. This protein actively transports bile salts from the liver into the bile canaliculus, ensuring normal bile flow through the osmotic process.^19^ Polymorphisms in *ABCB11* are associated with progressive familial intrahepatic cholestasis type 2 and an increased risk of HCC during early human development. Specifically, c.1331T>C (rs2287622) is a non-synonymous polymorphism (p.Val444Ala) located in exon 13 that is associated with cholestatic liver disease (mainly progressive intrahepatic cholestasis) and drug-induced cholestasis characterised by decreased or interrupted bile flow.^20^ ^,^ ^21^ ^,^ ^22^ ^,^ ^23^

Genetic elements that confer a greater predisposition to liver problems can act as important regulatory factors in worsening the outcomes of patients with chronic hepatitis, especially those with HCV infection. The presence of the 3435T, 2677T, and 1236T variant alleles in *ABCB1* can influence the plasma concentration and sustained virological response (SVR) of several HCV drugs, including telaprevir, sofosbuvir, and ribavirin (RBV).^24^ ^,^ ^25^ The homozygous TT genotype of the *ABCB1* polymorphism in exon 26 is associated with a 2.0-fold greater risk of non-response to drugs than the homozygous CC and heterozygous CT genotypes.^24^ The c.1331T>C SNP in *ABCB11* exerts an apparent influence on the pharmacokinetics of RBV and significantly elevates total bilirubin levels after antiviral therapy. The increase is greater in individuals with the 1331CC genotype than in those with 1331TT.^25^

Investigation of the influence of various *ABCB1* (c.1236C>T, c.2677G>T, and c.3435C>T) and *ABCB11* (c.1331T>C) SNPs will enhance our understanding of the clinical evolution of patients carrying the variants, since this variants can aggravate hepatic tissue damage and cause cholestasis in predisposed individuals, thereby increasing the levels of biochemical markers of liver injury that should be monitored. Thus, this study aimed to investigate the genotypic and allelic profiles of c.1236C>T, c.2677G>T, c.3435C>T, and c.1331T>C as well as their relationships with the clinical and laboratory characteristics of patients with chronic HCV infection unresponsive to interferon (IFN) and/or RBV treatment.

## SUBJECTS AND METHODS

*Ethical aspects* - This study was approved by the Ethics Committee of the Oswaldo Cruz Foundation (CAAE 34246914.4.1001.5248; number:2.927.747/18). The purpose of the study was explained to the participants. Confidentiality regarding patient identity and personal information was assured by highlighting that only researchers could access the information, which would be used solely for research purposes. The participants were then asked to sign an informed consent form.

*Study sample and clinical data* - In this retrospective, hypothesis-driven study, designed to investigate whether genetic variants in the *ABCB1* and *ABCB11* genes influence the disease progression in chronic HCV patients (primary hypothesis). For the purposes of this study, disease progression was defined as the presence of advanced fibrosis (grade F3) or cirrhosis (grade fibrosis F4), which constituted the primary outcome of the study. The secondary outcomes included laboratory and clinical parameters biologically linked to liver injury or metabolic processes potentially affected by ABCB gene polymorphisms, like lipid profile, liver enzymes, and metabolic biomarkers. Since the study includes both hypothesis-driven components (primary outcome) and exploratory components (secondary outcomes), we acknowledge the issue of multiple comparisons in the Discussion and clarify that exploratory associations should be interpreted with caution.

We analysed the medical records and laboratory test results of 232 patients with chronic HCV infection (genotypes 1a and 1b), pooled between November 2015 and November 2017 from the Liver Diseases Outpatient Clinic of Gaffrée and Guinle University Hospital (Federal University of the State of Rio de Janeiro — UNIRIO) in Rio de Janeiro, Brazil. All patients had been diagnosed with hepatitis as confirmed through serological and molecular tests. Clinical and laboratory information was obtained from the outpatient records and registered into our database by a specialised medical team.

The data obtained included age, sex, body mass index, diabetes status, serological levels of liver enzymes (AST, ALT, and GGT) and metabolic biomarkers [albumin, glucose, triglycerides, total cholesterol, low-density lipoprotein (LDL), HDL, bilirubin, alpha-fetoprotein (AFP)], cellular and haematological biomarkers (platelets, haemoglobin, leukocytes, and haematocrit), viral load, homeostatic model assessment of insulin resistance, hepatic steatosis, METAVIR activity and fibrosis/cirrhosis level, and histopathological examinations of liver biopsies. Reference values were adopted for each biomarker mentioned, following protocols described in the literature.^26^ ^,^ ^27^

A simple random probability sampling form was used to ensure that the study sample was representative of the overall population, thereby guaranteeing the internal validity of the study. The minimum number of participants (n = 57) was determined using the equation n = *z* ^2^ × p × (1 - p)/*e* ^2^, where *z* is the confidence level based on a standard normal distribution (1.96 for 95% confidence interval), p is the expected prevalence (0.183 for HCV in the general population, because no data were available for the study sample), and *e* is the maximum acceptable error in the estimate (0.05).^28^

The inclusion criteria were as follows: HCV-infected patients; older than 18 years of age; receiving follow-up care at the liver disease outpatient clinic; and having signed the informed consent form. The exclusion criteria were as follows: Patients co-infected with other hepatitis viruses (A and E); and haemolysed samples and/or samples with a volume of less than 200 μL.

*Nucleic acid extraction and analysis of the *ABCB1* and *ABCB11* genes* - The nucleic acid extraction from the serum sample was performed using the QIAamp RNA Mini Kit (QIAGEN, Hilden, Germany) according to the manufacturer's instructions. This extraction kit was selected due to its ability to simultaneously isolate DNA and RNA, allowing a single extraction step to be used for the analysis of HCV, an RNA virus, as reported in a previously published study,^29^ as well as for the analysis of human genes performed in the present study.

Genotyping of polymorphisms in the *ABCB1* and *ABCB11* genes was performed using real-time polymerase chain reaction (qPCR) with the TaqMan Universal PCR Master Reagent Mix (Applied Biosystems, Foster City, CA, United States) and the TaqMan SNP Genotyping Assay kit (Applied Biosystems) for allele discrimination for each polymorphism, according to the manufacturer's instructions.

The TaqMan SNP genotyping assays used for each polymorphism were as follows: *ABCB1* c.1236C>T (Assay ID: C___7586662_10), *ABCB1* c.2677G>T (Assay ID: C_11711720C_30), *ABCB1* c.3435C>T (Assay ID: C___7586657_20), and *ABCB11* c.1331T>C (Assay ID: C__16182459_10). Each assay involved a pair of oligonucleotides (sense and antisense) and a pair of fluorophores (VIC and FAM)-labelled probes with distinct excitation/emission spectra to distinguish the alleles. Three genotypes for each polymorphism were determined based on the manufacturer's information regarding the excitation and emission spectra of each fluorophore used in the TaqMan SNP genotyping assays [Supplementary data (Table I)].

*Statistical analysis* - All statistical analyses were performed using RStudio software (version 4.1.1, 2021). The significance level was set to a p-value of less than 0.05. Data are presented as frequencies and percentages for categorical variables and as means with standard deviations (SD) for continuous variables. The univariate analysis was performed using the chi-squared (χ²) test, Mann-Whitney *U* test, Student's *t*-test, or Wilcoxon's test to analyse categorical, parametric continuous, and non-parametric variables, as appropriate. Associations between individuals with genetic variants and those with wild-type genotype were evaluated against several potential predictor variables (body mass index, levels of fasting blood glucose, triglycerides, total cholesterol, LDL, HDL, AST, ALT, and GGT, degree of fibrosis, steatosis, and platelet count).

The comparisons between genotype groups and demographic variables (age, sex, BMI, diabetes, and others) were performed because these variables are well-established confounders in liver disease progression and in several secondary outcomes analysed in the study, including lipid profile, liver enzymes, and AFP levels. Assessing genotype distribution across these variables allows us to verify whether these factors may influence the associations observed and to ensure adequate characterisation of the cohort.

In the genotype-based analysis, individuals carrying the wild-type genotype were used as the reference group. Comparisons were performed separately between wild-type and heterozygous genotypes and between wild-type and mutant genotypes. For these analyses, continuous variables are presented as mean ± SD calculated based on the number of individuals in each genotype group. Heterozygous individuals were not duplicated within the same comparison. For allele-based analyses, the dataset was expanded to account for the two alleles carried by each individual, resulting in a total of 464 alleles. In this approach, continuous variables are presented as mean ± SD calculated based on allele counts, using alleles as the unit of analysis. Comparisons were performed between wild-type and variant alleles.

To determine whether the associations between the genetic polymorphisms and the clinical or laboratory parameters were independent of potential confounding factors, multivariate regression models were performed. For continuous outcomes, we employed multiple linear regression, whereas for categorical outcomes we used multinomial or logistic regression, as appropriate. The variables age, sex, BMI, and diabetes status were included as covariates in all multivariate models, as they are well-established predictors of lipid alterations, hepatic injury markers, and disease progression in chronic hepatitis C. These covariates were introduced simultaneously to adjust for their confounding effects and to estimate the independent contribution of each genotype or allele to the outcomes evaluated.

## RESULTS

*Clinical and epidemiological profiles* - The clinical and epidemiological profiles of the study participants are described in Table I and stratified by the studied polymorphisms genotypes in Supplementary data (Table II). The mean age of the patients was 61.2 ± 9.8 years, with the number of individuals increasing progressively with an increase in the age range, and approximately 56.9% were females. Additionally, 32.2% were overweight and 16.5% were obese. A large minority of patients (46.1%) had steatosis, and the majority (59.1%) had F4 fibrosis/cirrhosis, whereas the frequency of HCC was low (2.2%). Approximately 12.1% (28/232) of the patients were co-infected with HIV.

**TABLE I:** Clinical and epidemiological profiles of patients with chronic hepatitis C virus (HCV) infection

Variable	Value (n = 232)
Age, yr	61.19 ± 9.8
Sex	
Female	132 (56.9%)
Male	100 (43.1%)
Body mass index, kg/m^2^	26.38 ± 4.58
Diabetes	
Yes	66 (28.4%)
No	159 (68.5%)
ND	7 (3.0%)
Albumin, mg/dL	4.04 ± 0.7
Glucose, mg/dL	106.7 ± 30.0
Triglycerides, mg/dL	118.6 ± 145.9
Total cholesterol, mg/dL	161.4 ± 32.1
Low-density lipoprotein, mg/dL	90.14 ± 44.8
High-density lipoprotein, mg/dL	56.0 ± 44.2
Aspartate aminotransferase, U/L	62.59 ± 49.5
Alanine aminotransferase, U/L	62.27 ± 51.7
AST/ALT ratio	1.07 ± 0.6
Platelets/L	163.07 × 10^3^ ± 94.3 × 10^3^
Gamma-glutamyl transferase, U/L	102.10 ± 103.7
Total bilirubin, mg/dL	0.85 ± 0.5
Haemoglobin, g/L	13.22 ± 2.0
Leukocytes, cells/mm^3^	5752.0 ± 3667.3
Haematocrit	40.31% ± 5.0%
Alpha-fetoprotein, ng/mL	17.90 ± 30.1
Elastography, Kpa	18.82 ± 12.1
Steatosis	
Yes	107 (46.1%)
No	46 (19.8%)
ND	79 (34.0%)
Hepatocellular carcinoma	
Yes	5 (2.2%)
No	227 (97.8%)
Fibrosis	
F1	12 (5.1%)
F2	20 (8.6%)
F3	63 (27.2%)
F4 (Cirrhosis)	137 (59.0%)
HCV genotype	
1a	121 (52.2%)
1b	111 (47.8%)

AST: aspartate aminotransferase; ALT: alanine aminotransferase; ND: no data. Values are presented as n (%) or mean ± standard deviation (SD), as appropriate. Reference values: BMI: 18.5-25 = normal; 25-30 = overweight; > 30 = obesity; AST (U/L): 5-40 = normal; > 40 = elevated; ALT (U/L): 7-56 = normal; > 56 = elevated; GGT (U/L) male: 8-61 = normal; > 61 = elevated; GGT (U/L) female: 5-36 = normal; > 36 = elevated; Bilirubin (mg/dL): ≤ 1.2 = normal; Albumin (g/dL): 3.5-4.7 = normal; < 3.5 = low; > 4.7 = high; Cholesterol (mg/dL): ≤ 190 = normal; >190 = elevated; Platelets (1000/μL): 140-450 = normal; < 140 = thrombocytopenia; Glucose (mg/dL): ≤ 99 = normal; > 99 = elevated.^26^ ^,^ ^27^

*Frequency of polymorphisms* - With regard to the *ABCB1* SNPs (Figure), the frequencies of the CC, CT, and TT genotypes of c.1236C>T (rs1128503) were 49.6% (115/232), 32.2% (91/232), and 11.2% (26/232), respectively, whereas the frequency of the wild-type (C) allele was 69.2% and that of the variant allele (T) was 30.8%. For c.3435C>T (rs1045642), 15.5% (36/232) of the patients carried the TT genotype, 42.7% (99/232) carried the heterozygous genotype (CT), and 41.8% (97/232) carried the CC genotype. Moreover, the wild-type allele (C) is shown to be present in 63.2% of the samples and the variant allele (T) in 36.8%. For c.2677G>T (rs2032582), the frequency of the GG genotype was 55.6% (129/232), that of the TT genotype was 9.0% (21/232), and that of the heterozygous (GT) genotype was 35.4% (82/232), and the wild-type (G) and variant (T) alleles frequencies were 73.3% and 26.7%, respectively.

With regard to c.1331T>C (rs2287622) in the *ABCB11* gene (Figure), 54.3% (126/232) of the patients carried the heterozygous genotype (TC), 14.7% (34/232) had the TT genotype, and 31.0% (72/232) carried the CC genotype. The wild-type allele (T) was present in 41.8% of the patients and the variant allele (C) in 58.2%. All genotype frequencies for the studied polymorphisms were in Hardy–Weinberg equilibrium based on the expected genotype frequencies calculated [Supplementary data (Table III)].

**Figure:**
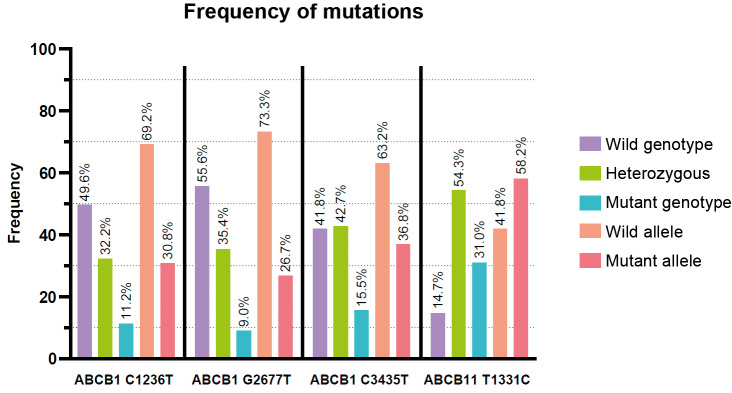
Frequencies of the polymorphisms studied in patients with chronic hepatitis C virus (HCV) infection. These genotyping results were generated using Quant Studio 3. The graph is separated according to single-nucleotide polymorphism (SNP) and gene (ATP-binding cassette subfamily B member 1 - *ABCB1*, or member 11 - *ABCB11*), with purple representing the wild-type genotype (1236CC, 2677GG, 3435CC, or 1331TT), green the heterozygous genotype (1236CT, 2677GT, 3435CT, or 1331TC), and blue the variant genotype (1236TT, 2677TT, 3435TT, or 1331CC). Orange represents the frequency of the wild-type allele (1236C, 2677G, 3435C, or 1331T) and pink the frequency of the variant allele (1236T, 2677T, 3435T, or 1331C).

*Association analysis* - The four SNPs were evaluated in the genotype-based analysis for their effects on all studied biochemical and epidemiological variables, and the results are presented in Supplementary data (Table I). Table II includes only the variables that showed statistically significant differences according to genotype-based or allele-based analysis. In addition, only the SNPs that presented significant associations are displayed in this table, specifically *ABCB1* 2677G>T, *ABCB1* 1236C>T, and *ABCB11* 1331T>C. Regarding the *ABCB1* c.3435C>T polymorphism, no statistically significant associations were observed with any of the clinical or laboratory parameters evaluated (data not shown).

In the univariable analysis (Table II), patients carrying the 2677TT genotype or 2677T allele had significantly lower levels of total cholesterol (p < 0.05) than those carrying the 2677GG or 2677GT genotypes or 2677G allele. Furthermore, significantly lower levels of HDL (p < 0.05) were associated with the 2677TT genotype and 2677T allele. In the multivariate analysis (Table II), a lower total cholesterol level continued to be observed among carriers of the 2677TT genotype [β = -32.16; 95% confidence interval (CI): -48.9 to -15.4; p < 0.01] and 2677T allele (β = -11.7; 95% CI: -19.2 to -4.3; p < 0.01). However, HDL levels were no longer significant (genotype: β = -16.6; 95%CI: -46.7 to 13.5; p > 0.1; allele: β = -3.01; 95% CI: -15.2 to 9.2; p > 0.1).

In the univariate and multivariate analysis, the total cholesterol level was significantly lower (p < 0.05) in carriers of the 1236TT genotype (β = -30.76; 95% CI: -45.5 to -15.9 p < 0.01) or 1236T allele (β = -11.9; 95% CI: -19.1 to -4.8; p < 0.01) than in individuals carrying 1236CC and 1236CT genotypes, or 1236C allele (Table II).

With regard to c.1331T>C, in the univariate analysis, the presence of the variant-type genotype (1331CC) was a risk factor for the development of liver cirrhosis (p < 0.05; OR: 2.40; 95% CI: 1.04-5.60) in this study samples compared with that of the wild genotype (1331TT). And carriers of the 1331CC genotype had higher AST levels (p < 0.05) than those in individuals carrying the 1331TT genotype (Table II). In the multivariate analysis, the association between the c.1331T>C variant and cirrhosis remained significant in the genotype-based analysis (adjusted OR = 3.05; 95% CI: 1.15 to 8.49), with the variant genotype showing a higher frequency of cirrhosis. Regarding AST levels, the association observed in the genotype-based analysis was no longer statistically significant after adjustment (β = 15.89; 95% CI: -3.9 to 35.8; p > 0.1), but in allele-based analysis the variant 1331C allele continued to show a significant association (β = 9.44; 95% CI: 0.73 to 18.2; p < 0.05) (Table II).

In the multivariate allele-based analysis (Table II), a positive diabetes status was independently associated with cholesterol levels, whereas sex and BMI were related to AST levels (p < 0.05). These findings highlight the importance of both genetic factors and biochemical/clinical variables as independent contributors to metabolic alterations.

**TABLE II:** Statistical analysis of significant variables associated with the single-nucleotide polymorphisms (SNPs) c.2677G>T and c.1236C>T in the ATP-binding cassette subfamily B member 1 (*ABCB1*) gene and c.1331T>C in the *ABCB11* gene

Analysis by genotype				p-value (univariate)	Adjusted p-value (multivariate)	Analysis by allele		p-value (univariate)	Adjusted p-value (multivariate)
*ABCB1* c.2677G>T SNP									
	GG (n = 129)	GT (n = 82)	TT (n = 21)			2677G (n = 339)	2677T (n = 125)		
Total cholesterol, mg/dL	166.7 ± 31.1	159.2 ± 30.4	139.1 ± 35.8	**< 0.01**	**< 0.001** (β = -32.16; 95% CI: -48.9 to -15.9)	164.73 ± 30.9	152.68 ± 33.4	**< 0.01**	**<0.01** (β = -11.7; 95% CI: -19.2 to -4.3)
High-density lipoprotein, mg/dL	57.0 ± 29.3	57.3 ± 62.1	42.0 ± 14.6	**< 0.05**	0.27 (β = -16.6; 95% CI: -46.7 to 13.5)	57.10 ± 40.3	52.91 ± 53.4	**< 0.01**	0.62 (β = -3.01; 95% CI: -15.2 to 9.2)
*ABCB1* c.1236C>T SNP									
	CC (n = 115)	CT (n = 91)	TT (n = 26)			1236C (n = 321)	1236T (n = 143)		
Total cholesterol, mg/dL	165.9 ± 30.1	162.5 ± 30.0	137.1 ± 38.7	**< 0.001**	**< 0.001** (β = -30.76; 95% CI: -45.5 to -15.9)	164.87 ± 30.0	153.70 ± 35.1	**< 0.01**	**<0.01** (β = -11.9; 95% CI: -19.1 to -4.8)
***ABCB11* c.1331T>C SNP**									
	TT (n = 34)	TC (n = 126)	CC (n = 72)			1331T (n = 194)	1331C (n = 270)		
Cirrhosis				**< 0.05** (OR: 2.40; 95% CI: 1.04–5.60)	**< 0.05** (adjusted OR = 3.05; 95% CI: 1.15 to 8.49)			**0.05**	0.1 (adjusted OR = 1.51; 95% CI: 0.98 to 2.34)
Yes	16 (47.1)	72 (57.1)	49 (68.1)			90 (46.4)	100 (37.0)		
No	18 (52.9)	54 (42.9)	23 (31.9)			104 (53.6)	170 (63.0)		
Aspartate aminotransferase, U/L	59.6 ± 32.6	54.8 ± 37.8	77.9 ± 68.1	**< 0.05**	0.11 (β = 15.89; 95% CI: -3.9 to 35.8)	56.39 ± 36.0	67.02 ± 56.8	0.2	**< 0.05** (β = 9.44; 95% CI: 0.73 to 18.2)

*ABCB1*: ATP-binding cassette subfamily B member 1; *ABCB11*: ATP-binding cassette subfamily B member 11; CI: confidence interval; SNP: single-nucleotide polymorphism. The statistical analysis was performed by genotype and by allele for each polymorphism studied. Continuous variables are presented as mean ± standard deviation (SD). In genotype-based analyses, values were calculated per individual within each genotype group, using the wild-type genotype as reference. In allele-based analyses, values were calculated using allele counts as the unit of analysis. No significant results were found for the analysis of the c.3435C>T polymorphism; therefore, this SNP was not included in Table II. In the multivariate analysis, potential confounders (age, sex, body mass index, and diabetes) were incorporated into the model to control for their effects. *In the multivariate allele analysis, a positive diabetes status was independently associated with cholesterol levels, whereas sex and body mass index (BMI) were related to aspartate aminotransferase (AST) levels (p < 0.05).

In the univariate combined allele analysis (Table III), the presence of the fully variant combined allele (1236T, 2677T, 3435T, and 1331C; TTTC) was significantly associated with lower total cholesterol and higher AFP levels compared to other genotypes (p < 0.05). However, in the multivariate analysis, only the lower cholesterol levels (β = -10.84; 95% CI: -19.25 to -2.4; p < 0.05) remained associated with the fully variant combined allele (Table III). In addition, in the combined-allele multivariate analysis (Table III), cholesterol levels were also independently associated with diabetes, reinforcing the importance of both variables as independent contributors to metabolic alterations.

**TABLE III:** Analysis by combining allele of the studied variants

Analysis by combined allele			p-value (univariate)	Adjusted p-value (multivariate)
	Fully variant combined allele TTTC (n = 88)	Other allelic combination (n = 376)		
Alpha-fetoprotein, ng/mL	19.07 ± 22.1	17.63 ± 31.5	**< 0.05**	0.69 (β = 2.23; 95% CI: -8.9 to 13.40)
Total cholesterol, mg/dL	153.33 ± 31.1	163.29 ± 32.1	**< 0.05**	**< 0.05** (β = -10.84; 95% CI: -19.2 to -2.4)

CI: confidence interval. The analysis was carried out by stratifying the 16 possible combined alleles into two groups: (1) alleles carrying the variant form for all four polymorphisms studied, or fully variant combined allele (1236T and 2677T and 3435T and 1331C; TTTC); and (2) composed of the remaining possibilities, that is, alleles presenting at least one wild-type allele among the four loci (1236C or 2677G or 3435C or 1331T). In the multivariate analysis, potential confounders (age, sex, body mass index - BMI, and diabetes) were incorporated into the model to control for their effects. *In the combined-genotype multivariate analysis, cholesterol levels were also independently associated with diabetes.

## DISCUSSION

This study highlights the impact of *ABCB1* and *ABCB11* gene polymorphisms in patients with chronic HCV infection unresponsive to IFN and/or RBV therapy. The presence of polymorphism in *ABCB1* (c.2677G>T, c.3435C>T, and c.1236C>T) and *ABCB11* (c.1331T>C) is known to be associated with drug resistance, hepatotoxicity, and cholestasis as well as resistance to drugs used in the treatment of HCV-infected patients, such as telaprevir, sofosbuvir, and RBV.^15^ ^,^ ^16^ ^,^ ^17^ ^,^ ^20^ ^-^ ^25^ In this study, 59.0% of the patients had advanced liver diseases such as cirrhosis (F4) and 2.2% had HCC. Some studies have shown that the presence of these polymorphisms can directly or indirectly influence the clinical evolution of HCV-infected patients to cirrhosis and HCC.^30^ ^,^ ^31^ ^,^ ^32^ ^,^ ^33^ ^,^ ^34^

Our results showed that, among *ABCB1* polymorphisms, the 2677TT genotype occurred at the lowest frequency, being present in only 9.0% of the samples, whereas the 1236TT and 3435TT genotypes were observed in 10.8% and 15.5% of the cohort, respectively. In contrast, the 1331CC genotype of the *ABCB11* was found in 31.0% of the samples. Overall, in the genotype-based analysis for these four polymorphisms, we observed a greater prevalence of heterozygote genotypes in the study samples, which may be a characteristic of a mixed population such as that in Brazil. All variants were in Hardy-Weinberg equilibrium, indicating that the observed genotype frequencies were consistent with those expected for a genetically stable sample. This supports the reliability of the genotyping procedures and suggests that no sample stratification or methodological bias significantly affected the distribution of these genotypes, thereby reinforcing the validity of the association analyses performed.

The study was primarily hypothesis-driven, focused on evaluating whether *ABCB1* and *ABCB11* variants influence the development of advanced fibrosis or cirrhosis. The assessment of secondary biochemical and metabolic parameters reflects the biological relevance of *ABC* transporters in hepatic injury and bile acid regulation. However, we recognise that the substantial number of variables included also introduces exploratory elements. Therefore, the potential for type I error is acknowledged, and the secondary findings should be interpreted as hypothesis-generating.

It was observed, in the univariate and multivariate genotype or allele-based analysis, that the 2677TT and 1236TT genotypes were an independent factor significantly associated with lower total cholesterol levels compared with their wild-type and heterozygous genotypes. Additionally, the presence of the variant allele (T) in the 2677 and 1236 polymorphisms correlated with lower serum levels of total cholesterol compared with the levels in patients with the wild-type alleles (2677G and 1236C). The worsening of HCV-associated liver disease is related to changes in the lipid profile, with significant reductions in total cholesterol, as the liver is responsible for lipid metabolism.^5^ ^,^ ^6^ ^,^ ^7^ These changes in the lipid profile are usually mainly observed in individuals with decompensated cirrhosis and HCC. However, because the patients in this study had predominately compensated cirrhosis, the lipid profile decrease was unexpected. Therefore, the reductions in total cholesterol in these individuals may be closely related to the presence of 2677TT and 1236TT genotypes, which may be associated with a worse prognosis and a greater risk of developing cirrhosis and liver cancer.

In the univariate genotype and allele-based analysis, the 2677TT genotype and 2677T allele were associated with a lower serum HDL level, but not in the multivariate. The disappearance of the association in the adjusted analysis may reflect the stronger influence of metabolic comorbidities, such as diabetes, on lipid parameters, which can mask or override the modest genetic effect expected for this polymorphism.

In univariate and multivariate analysis of our cohort, the presence of the variant-type 1331CC genotype in *ABCB11* was a risk factor for the development of liver cirrhosis (p < 0.05) as compared with the presence of 1331TT genotype, indicating that the variant genotype is a relevant factor in the development of advanced liver disease in patients with chronic HCV infection. Carriers of the 1331CC genotype also exhibited higher AST levels (p < 0.05) compared with individuals carrying 1331TT or 1331TC genotypes. In the multivariate allele analysis, the 1331T>C variant remained independently associated with AST levels. AST is an important biomarker of liver injury, with its higher serum levels indicating greater tissue damage.

The importance of the 1331CC genotype as a risk factor for progression to cirrhosis under chronic HCV infection is supported by other research findings, where its presence has been shown to increase plasma bile acid levels, consequently worsening fibrosis and increasing the risk of HCC in HCV-infected individuals.^31^ ^,^ ^32^ ^,^ ^33^ Additionally, in our exploratory analysis [Supplementary data (Table II)], we observed higher levels of GGT in individuals carrying 1331CC genotype than in those carrying the wild-type genotype, although the difference was not statistically significant. Increased expression of GGT, an important canalicular membrane enzyme and a biomarker of liver damage, is associated with worse prognosis and reduced survival in patients with HCC.^35^

In the exploratory analysis of clinical data [Supplementary data (Table II)], no clear pattern was detected among the polymorphisms. For *ABCB1* c.2677G>T, higher AST, ALT, and bilirubin levels were observed mainly in individuals carrying the TT genotype (and, for ALT and bilirubin, also in GT carriers). In contrast, for *ABCB1* c.1236C>T and c.3435C>T, lower AST and ALT levels were observed among CT and TT genotypes, while bilirubin levels tended to be lower in CT and higher in TT genotype carriers. For *ABCB11* c.1331T>C, AST and ALT levels were lower in TC genotype carriers but higher in CC genotype, whereas bilirubin levels were higher in both TC and CC genotypes. However, these differences did not reach statistical significance, possibly because most of patients in the cohort had cirrhosis, a condition that independently elevates liver injury markers and may confound genotype-related effects. In addition, a higher frequency of HCC was observed among carriers of variant genotypes relative to wild-type individuals; nevertheless, given the small number of HCC cases in the sample (2.2%), this observation should be interpreted with caution and does not allow for definitive conclusions.

For the analysis by combined alleles, the alleles profile was categorised into two groups to optimise and facilitate understanding of the statistical analysis results, since there were 16 combinations. The first group comprised alleles carrying the variant form at all four loci (1236T and 2677T and 3435T and 1331C; TTTC); called of fully variant combined allele. The second group comprised the other possible combination of alleles; that is, alleles carrying at least one wild-type allele (1236C or 2677G or 3435C or 1331T) among the four polymorphisms studied. Consequently, we observed that the presence of the variant TTTC combined allele was significantly associated with a lower level of total cholesterol compared with the effects of the other alleles, in univariate and multivariate analysis. Corroborating the other results of this study that showed the importance of these polymorphisms as independent factors in the expression of the lipid profile and consequently the clinical evolution of patients with chronic HCV infection.

Moreover, we observed a higher serum level of AFP in the group with the fully variant combined allele (TTTC) in the univariate analysis, however, this association did not remain significant after adjustment for clinical confounders in the multivariate model. AFP is an important biomarker for diagnosing and monitoring the emergence of tumours, especially HCC. In patients with chronic HCV infection, especially with advanced stages of fibrosis/cirrhosis (present in 59.0% of our study group), the presence of elevated AFP may mean a greater risk of developing HCC, even after achieving SVR.^36^ However, the loss of significance after adjustment suggests that the variant allele alone may not independently influence AFP levels, and that the observed elevation is more likely explained by the burden of liver disease rather than by a direct genetic effect. Nonetheless, the descriptive trend observed in the univariate analysis highlights the need for future studies with larger sample sizes and better stratification by fibrosis stage to further clarify whether specific genetic profiles could modulate AFP dynamics in chronic HCV infection.

This study highlights the roles of *ABCB1* and *ABCB11* polymorphisms in liver disease progression in patients with chronic HCV infection. The presence of these four SNPs was either directly (1331TT genotype) or indirectly (via lower total cholesterol and HDL and higher AST and AFP levels) related to the development of liver cirrhosis, which is a risk factor for HCC.

Cholestasis is characterised by the decrease or interruption of bile flow to the duodenum, where changes in alkaline phosphatase, GGT, and bilirubin levels can be observed.^19^ HCV infection can lead to intrahepatic cholestasis, and the presence of the c.1331T>C SNP is also a risk factor associated with several cholestatic diseases.^19^ ^,^ ^20^ ^,^ ^21^ ^,^ ^22^ ^,^ ^23^ In our study, we observed that individuals with the 1331CC variant genotype had higher levels of GGT and total bilirubin than those carrying the wild-type genotype 1331TT, albeit the difference was not statistically significant [Supplementary data (Table I)].

After the sample collection period, the patients received direct-acting antivirals (DAAs), and 99.3% achieved SVR. This high SVR rate suggests that the studied *ABCB1* and *ABCB11* genetic polymorphisms were not associated with impaired treatment response in the era of interferon-free regimens. From a public health perspective, broad access to DAA therapy must be implemented as a key strategy for achieving HCV elimination. However, although DAAs have been available since 2014, ensuring universal access for all HCV-infected individuals remains a major challenge, particularly in low- and middle-income countries without universal healthcare policies, due to the high cost of these medications. In addition, the challenge of HCV elimination also depends on expanded access to diagnostic testing, especially early diagnosis. Currently, most individuals are diagnosed only decades after infection, when the liver is already compromised with advanced fibrosis or cirrhosis.

Finally, these results support the well-established stability of SVR with DAA therapy, regardless of host-related factors, reinforcing the need to expand access to INF-free regimens independently of the genetic background of infected individuals. It is also important to emphasise that achieving SVR reduces but does not eliminate the risk of developing HCC in patients with advanced liver disease (fibrosis F3 and cirrhosis), given that chronic inflammation and tissue damage are already established. Therefore, continued clinical surveillance remains essential even after the SVR. In this context, the presence of *ABCB1* and *ABCB11* polymorphisms may contribute to this clinical outcome; however, further studies are needed to evaluate their impact in the post-SVR setting under DAA therapy.

One limitation of this study was the older age of patients. Given that the participants were unresponsive to conventional treatment (IFN and/or RBV), many would have been infected with HCV for many years. Thus, the results reflected the effects on an older cohort and may be different in a younger cohort. Moreover, because of this long period of chronic HCV infection, most patients had advanced liver diseases, such as grade 3 fibrosis and cirrhosis. Consequently, this limited our knowledge regarding these SNPs in individuals with mild or moderate fibrosis since the presence of these polymorphisms can influence faster disease evolution. In addition, due to the large number of variables analysed, our findings should be interpreted as hypothesis-generating and require confirmation in future studies with greater statistical power.

Finally, we recommended that the patients with advanced fibrosis and cirrhosis be stringently monitored, even after DAA treatment, owing to the risk of developing HCC despite achieving SVR. However, many of the individuals had discontinued follow-up with the medical team, mainly because of the Coronavirus disease 19 (COVID-19) pandemic that occurred between 2020 and 2023. This situation has made it challenging to access further information on their post-treatment clinical evolution.

*In conclusion* - Studies have shown that polymorphisms in the *ABCB1* gene are associated with an increased risk of HCC and may predict significant changes in the pathological characteristics of the disease and influence patient response to anticancer treatment.^37^ ^,^ ^38^ ^,^ ^39^ Polymorphisms in the *ABCB11* gene can cause intrahepatic cholestasis and increased inflammation, negatively impacting liver cancer prognosis.^40^ However, there is no consensus regarding the relationship between these polymorphisms and HCC.^41^ ^,^ ^42^ Further studies are needed, especially with a focus on chronic HCV infection, because an SVR with DAAs does not exclude the risk of HCC in individuals with advanced cirrhosis, which must be continuously monitored.

To the best of our knowledge, this is the first study to evaluate the influence of *ABCB1* and *ABCB11* polymorphisms on the clinical and laboratory characteristics of patients with chronic HCV infection in Brazil. Given the known association of these SNPs with cholestatic damage and their impact on the efficacy and safety of antiviral drugs, it is important to establish their associated risks in the context of chronic HCV infection to understand their influence on the clinical evolution and clinical features of affected patients. Moreover, these genetic variants may serve as prognostic factors of liver damage.

## SUPPLEMENTARY MATERIALS

Supplementary material

## Data Availability

The datasets generated and/or analysed during the current study cannot be made publicly available due to ethical and privacy restrictions involving human participants. However, the data is available from the corresponding author upon reasonable request.
