# Supplementary material for: Association of p-glycoprotein and bile salt export pump gene polymorphisms with advanced liver disease in hepatitis C virus infected patients
Source: Mem Inst Oswaldo Cruz. 2026 Jul 20;121:e250173. doi: 10.1590/0074-02760250173 (PMC13387806; doi:10.1590/0074-02760250173)
Supplement: Supplementary material [file 1678-8060-mioc-121-e250173-s1.pdf]

TABLE I

Studied polymorphisms and expected genotypes

Polymorphism	Reference SNP (rs)	Assay ID	Genotype		
			Homozygous for allele 1 (VIC)	Heterozygous (VIC and FAM)	Homozygous for allele 2 (FAM)
ABCB1 c.2677G>T	rs2032582	C_11711720C_30	GG	GT	TT
ABCB1 c.3435C>T	rs1045642	C___7586657_20	TT	TC	CC
ABCB1 c.1236C>T	rs1128503	C___7586662_10	TT	TC	CC
ABCB11 c.1331T>C	rs2287622	C__16182459_10	CC	CT	TT

SNP: single-nucleotide polymorphism.

TABLE II

Clinical and epidemiological profile of individuals with hepatitis C virus (HCV) included in this study stratified by polymorphisms in their ABCB1 and ABCB11 genes

	ABCB1												*ABCB11*			
	G2677T				C3435T				C1236T				T1331C			
	GG N = 129	GT N = 82	TT N = 21	p-value	CC N = 97	CT N = 99	TT N = 36	p-value	CC N = 115	CT N = 91	TT N = 26	p-value	TT N = 34	TC N = 126	CC N = 72	p-value
Age, years	61.3 ± 10.0	60.1 ± 9.8	64.3 ± 8.6	0.25	61.7 ± 9.9	60.0 ± 9.7	63.1 ± 9.8	0.22	62.2 ± 9.8	59.4 ± 9.7	63.0 ± 9.7	0.09	59.7 ± 8.4	60.6 ± 9.9	63.0 ± 10.1	0.08
Gender				0.12				0.29				**0.04**				0.58
Female	79 (61.2%)	45 (54.9%)	8 (38.1%)		60 (61.9%)	55 (55.6%)	17 (47.2%)		67 (58.3%)	56 (61.5%)	9 (34.6%)		22 (64.7%)	69 (54.8%)	41 (56.9%)	
Male	50 (38.8%)	37 (45.1%)	13 (61.9%)		37 (38.1%)	44 (44.4%)	19 (52.8%)		48 (41.7%)	35 (38.5%)	17 (65.6%)		12 (35.3%)	57 (45.2%)	31 (43.1%)	
BMI, kg/m^2	26.6 ± 4.4	26.1 ± 5.0	26.4 ± 4.4	0.67	26.4 ± 4.4	26.4 ± 4.9	26.3 ± 4.5	0.90	26.5 ± 4.6	26.0 ± 4.7	27.0 ± 4.6	0.83	26.3 ± 3.0	26.5 ± 4.9	26.2 ± 4.6	0.95
Diabetes				0.30				0.88				0.90				0.87
Yes	34 (26.4%)	28 (34.1%)	4 (19.0%)		27 (27.8%)	29 (29.3%)	10 (27.8%)		33 (28.7%)	26 (28.6%)	7 (26.9%)		10 (29.4%)	36 (28.6%)	20 (27.8%)	
No	92 (71.3%)	50 (61.0%)	17 (81.0%)		68 (70.1%)	67 (67.7%)	24 (66.7%)		78 (67.8%)	62 (68.1%)	19 (73.1%)		22 (64.7%)	87 (69.0%)	50 (69.4%)	
ND	3 (2.2%)	4 (4.9%)	0 (0.0%)		2 (2.1%)	3 (3.0%)	2 (5.6%)		4 (3.5%)	3 (3.3%)	0 (0.0%)		2 (5.9%)	3 (2.4%)	2 (2.8%)	
Albumin, mg/dL	4.08 ± 0.8	4.03 ± 0.6	3.80 ± 0.6	0.12	4.12 ± 0.9	4.00 ± 0.6	3.93 ± 0.5	0.43	4.13 ± 0.8	3.97 ± 0.8	3.90 ± 0.6	0.25	4.14 ± 0.7	4.05 ± 0.8	4.00 ± 0.6	0.13
Glycose, mg/dL	105.4 ± 27.6	109.5 ± 35.6	103.8 ± 19.3	0.91	108.1 ± 30.8	106.1 ± 32.9	104.4 ± 16.7	0.53	107.1 ± 28.9	107.2 ± 33.8	103.2 ± 19.1	0.88	112.5 ± 40.6	105.2 ± 27.9	106.8 ± 28.2	0.76
Triglycerides, mg/dL	121.9 ± 189.3	115.1 ±66.4	112.4 ±57.8	0.42	135.5 ± 216.0	105.6 ± 61.7	110.0 ± 47.9	0.56	125.4 ± 200.5	114.8 ± 64.3	101.2 ± 47.5	0.37	171.7 ± 350.5	113.7 ± 64.5	101.0 ± 40.5	0.37
Cholesterol Total, mg/dL	166.7 ±31.1	159.2 ± 30.4	139.1 ± 35.8	**0.01**	163.5 ± 31.7	162.2 ± 32.5	153.6 v 32.0	0.31	165.9 ± 30.1	162.5 ± 30.0	137.1 ± 38.7	**0.01**	170.8 ± 38.4	158.4 ± 31.2	162.0 ± 29.8	0.16
LDL, mg/dL	89.9 ± 32.6	93.7 ± 58.8	73.4 ±30.8	0.16	89.1 v 32.9	93.4 ± 56.7	82.2 ± 29.5	0.57	90.1 ± 32.7	87.7 ± 26.7	101.3 ± 114.4	0.40	95.4 ± 38.8	85.9 ± 27.2	94.2 ± 64.7	0.86
HDL, mg/dL	57.0 ±29.3	57.3 ±62.2	42.0 ± 14.6	**0.02**	54.0 ± 32.3	59.1 ± 57.3	51.7 ± 18.9	0.45	53.4 ± 15.8	55.3 ± 31.7	73.1 ± 127.1	0.16	49.8 ± 15.1	53.4 ± 30.8	63.1 ± 65.9	0.20
AST, U/L	63.5 ± 57.4	58.5 ± 36.6	66.7 ± 41.1	0.60	71.2 ± 63.9	52.6 ± 31.6	67.1 ± 40.4	0.08	66.7 ± 60.9	57.0 ± 32.9	63.5 ± 41.1	0.86	59.4 ± 32.6	54.8 ± 37.8	77.9 ± 68.1	**0.04**
ALT, U/L	60.1 ± 48.5	64.2 ± 58.0	68.0 ± 46.2	0.48	66.0 ± 53.8	57.8 ± 50.9	65.4 ± 48.2	0.68	63.0 ±51.3	61.6 ± 55.6	61.5 ± 39.9	0.82	67.6 ± 53.0	57.1 ± 50.1	69.0 ± 53.6	0.14
AST/ALT ratio	1.04 ± 0.5	1.12 ± 0.7	1.05 ± 0.3	0.51	1.06 ± 0.5	1.11 ± 0.6	1.00 ± 0.4	0.58	1.05 ± 0.5	1.11 ± 0.6	1.04 ± 0.3	0.40	1.09 ± 0.4	1.07 ± 0.6	1.06 ± 0.5	0.59
Plaquettes/L	168.7 x 10^3 ± 109.1 x 10^3	156.6 x 10^3 ± 71.3 x 10^3	153.4 x 10^3 ± 73.9 x 10^3	0.91	162.2 x 10^3 ± 110.3 x 10^3	167.3 x 10^3 ± 82.1 x 10^3	153.1 x 10^3 ± 77.9 x 10^3	0.46	161.6 x 10^3 ± 97.4 x 10^3	163.9 x 10^3 ± 95.8 x 10^3	166.6 x 10^3 ± 75.5 x 10^3	0.76	157.9 x 10^3 ± 77.4 x 10^3	161.8 x 10^3 ± 97.2 x 10^3	167.6 x 10^3 ± 97.3 x 10^3	0.87
GGT, U/L	103.6 ± 98.2	106.6 ± 117.1	75.6 ± 78.5	0.54	112.4 ± 103.0	98.1 ± 109.1	84.7 ± 88.4	0.18	105.6 ± 100.4	104.5 ± 114.4	78.3 ± 76.2	0.56	89.0 ± 77.2	99.2 ± 107.9	113.4 ± 107.1	0.38
Bilirubin total, mg/dL	0.82 ± 0.5	0.83 ± 0.6	1.03 ± 0.7	0.58	0.84 ± 0.5	0.82 ± 0.5	0.93 ± 0.7	0.80	0.85 ± 0.5	0.82 ± 0.6	0.88 ± 0.6	0.40	0.78 ± 0.3	0.86 ± 0.6	0.84 ± 0.5	0.97
Haemoglobin, g/L	13.0 ± 1.9	13.3 ± 2.1	14.0 ± 1.9	0.22	13.0 ± 2.1	13.2 ± 2.0	14.0 ± 1.6	0.09	13.1 ± 1.9	13.2 ± 2.0	13.8 ± 2.4	0.38	13.0 ± 2.5	13.2 ± 2.0	13.3 ± 1.8	0.95
Leucocytes, cells/mm^3	5901.9 ± 4618.8	5489.2 ± 1866.8	5862.0 ± 2069.9	0.65	6045.8 ± 5170.5	5534.3 ± 1920	5533.8 ± 1869.7	0.81	5838.7 ± 4848.6	5503.7 ± 1953.2	6290.8 ± 2027.6	0.10	5850.4 ± 1785.5	5271.8 ± 1882.9	6537.6 ± 5885.5	0.16
Haematocrit	40.0% ± 5.1 %	40.4% ± 5.5%	41.0 ± 5.1%	0.58	40.2% ± 4.9%	39.9% ± 5.7%	41.6% ± 5.1%	0.49	40.3% ± 5.4%	40.0% ± 4.0%	41.2% ± 6.3%	0.55	40.8% ± 5.7%	40.3% ± 5.6%	40.1% ± 4.5%	0.62
Alpha-fetoprotein, ng/mL	18.8 ± 35.9	17.6 ± 22.9	14.0 ± 14.9	0.21	14.2 ± 29.5	18.8 ± 27.6	27.9 ± 39.8	0.06	19.9 ± 37.9	15.0 ± 18.0	19.1 ± 27.4	0.83	19.8 ± 35.7	17.9 ± 29.8	17.2 ± 29.3	0.74
Elastography, Kpa	19.2 ± 12.2	17.7 ± 9.0	21.2 ± 20.3		18.6 ± 11.0	18.2 ± 10.9	21.0 ± 17.0		18.9 ± 11.8	18.4 ± 10.3	19.7 ± 18.4		19.3 ± 15.7	19.4 ± 12.5	17.7 ± 9.4	
Steatosis				0.38				0.92				0.39				0.29
Yes	62 (48.1%)	36 (43.9%)	9 (42.9%)		48 (49.5%)	44 (44.4%)	15 (41.7%)		57 (49.6%)	39 (42.9%)	11 (42.3%)		15 (44.1%)	52 (41.3%)	40 (55.6%)	
No	29 (23.0%)	15 (18.3%)	2 (9.5%)		18 (18.5%)	20 (20.2%)	8 (22.2%)		25 (21.7%)	18 (19.8%)	3 (11.5%)		9 (26.5%)	26 (20.6%)	11 (15.3%)	
ND	38 (29.5%)	31 (37.8%)	10 (47.6%)		31 (32.0%)	35 (35.4%)	13 (36.1%)		33 (28.7%)	34 (37.4%)	12 (46.2%)		10 (29.4%)	48 (38.1%)	21 (29.2%)	
CHC				0.26				0.60				0.09				0.31
Yes	1 (0.8%)	3 (3.7%)	1 (4.8%)		1 (1.0%)	3 (3.0%)	1 (2.8%)		1 (0.9%)	2 (2.2%)	2 (7.7%)		0 (0.0%)	2 (1.6%)	3 (4.2%)	
No	128 (99.2%)	79 (96.3%)	20 (95.2%)		96 (99.0%)	96 (97.0%)	35 (97.2%)		114 (99.1%)	89 (97.8%)	24 (92.3%)		34 (100.0%)	124 (98.4%)	69 (95.8%)	
Fibrosis				0.56				0.98				0.54				**0.04**
F1	9 (7.0%)	3 (3.7%)	0 (0.0%)		6 (6.2%)	5 (5.0%)	1 (2.8%)		7 (6.1%)	5 (5.5%)	0 (0.0%)		3 (8.8%)	4 (3.2%)	5 (6.9%)	
F2	13 (10.1%)	6 (7.3%)	1 (4.8%)		8 (8.2%)	9 (9.1%)	3 (8.3%)		13 (11.3%)	6 (6.6%)	1 (3.8%)		3 (8.8%)	15 (11.9%)	2 (2.8%)	
F3	30 (23.2%)	26 (31.7%)	7 (33.3%)		25 (25.8%)	27 (27.3%)	11 (30.6%)		27 (23.5%)	27 (29.7%)	9 (34.6%)		12 (35.3%)	35 (27.8%)	16 (22.2%)	
F4 (Cirrhosis)	77 (59.7%)	47 (53.7%)	13 (61.9%)		58 (59.8%)	58 (58.6%)	21 (58.3%)		68 (59.1%)	53 (58.2%)	16 (61.5%)		16 (47.1%)	72 (57.1)	49 (68.1%)	
HCV genotype				0.88				0.32				0.58				0.06
1a	67 (51.9%)	44 (53.7%)	10 (47.6%)		45 (46.4%)	56 (56.6%)	20 (55.6%)		58 (50.4%)	51 (56.0%)	12 (46.2%)		24 (70.6%)	64 (50.8%)	33 (45.8%)	
1b	62 (48.1%)	38 (46.3%)	11 (52.4%)		52 (53.6%)	43 (43.4%)	16 (44.4%)		57 (49.6%)	40 (44.0%)	14 (53.8%)		10 (29.4%)	62 (49.2%)	39 (54.2%)	

Values are presented as n (%) or mean ± standard deviation (SD), as appropriate. In genotype-based analyses, values were calculated per individual within each genotype group, using the wild-type genotype as reference. In allele-based analyses, values were calculated using allele counts as the unit of analysis. Reference values: BMI (body mass index) - Normal: 18.5-25, overweight: 25-30, obesity > 30; AST (U/L)- 5-40: normal, > 40: elevated; ALT (U/L)- 7-56: normal, > 56: elevated; GGT (U/L) - male 8-61: normal, > 61: elevated, female 5-36: normal, > 36 elevated; Bilirubin (mg/dL) - up to 1.2 l: normal; Albumin (g/dL) - 3.5-4.7: normal, < 3.5: low, > 4.7 high; Cholesterol (mg/dL)- up to 190 normal, > 190: high; Platelets (one thousand/**μL**) - 140-450: normal, < 140: Thrombocytopenia; Glucose (mg/dL)- up to 99: normal, > 99: elevated.[26,27]

TABLE III

Comparison between expected and obtained genotype frequencies of the studied polymorphisms in the study sample

Polymorphism		Expected absolute genotype count (Hardy-Weinberg)	Obtained absolute genotype count in the study	X^2	p-value	Conclusion
c.1236C>T	CC	111.04	115	1.490	0.22	In equilibrium
	CT	98.93	91			
	TT	22.04	26			
c.2677 G>T	GG	124.57	129	2.207	0.13	In equilibrium
	GT	90.86	82			
	TT	16.57	21			
c.3435C>T	CC	92.51	97	1.604	0.20	In equilibrium
	CT	107.98	99			
	TT	31.51	36			
c.1331T>C	TT	40.56	34	3.129	0.07	In equilibrium
	TC	112.89	126			
	CC	78.56	72			
