# Supplementary material for: Association of p-glycoprotein and bile salt export pump gene polymorphisms with advanced liver disease in hepatitis C virus infected patients
Source: Mem Inst Oswaldo Cruz. 2026 Jul 20;121:e250173. doi: 10.1590/0074-02760250173 (PMC13387806; doi:10.1590/0074-02760250173)
Supplement: Peer review history

# Reviewer report: FIRST REVIEW ROUND - REVIEWERS' COMMENTS

## REVIEWER #1

a) Adequacy of the abstract;

Considering the elements highlighted in the abstract's structure, the content appears appropriate and accurately reflects the methodology and results described in the study

b) Originality and importance of the contribution for the development of the field of study. And relevance of:

Regarding originality, I note that the topic has been widely discussed in literature, particularly in relation to genetic polymorphisms in ABC genes and their impact on Hepatitis C severity. However, this study brings a relevant contribution by analyzing a Brazilian cohort and correlating the polymorphism data with clinical and laboratory findings. The results are thoroughly discussed, including the study's limitations. I consider this work relevant and of interest to the field.

c) Methodology, results and discussion;

In terms of methodology, the study presents a well-defined and clearly described approach. The results of the polymorphism analysis using the PCR technique are thoroughly explored, both in the narrative and in the accompanying figures.

The discussion of the results is well developed, as it effectively correlates the data obtained from the different haplotypes evaluated in the study with the laboratory findings related to lipid profiles and hepatic parameters, including alpha-fetoprotein as a biomarker for hepatocellular carcinoma.

However, in the discussion of the results, it is not clear why the ABCB1 gene SNP -3435C>T was not included in Table IV. If no significant difference was observed, this should have been explicitly stated in the text. I also believe that the data presented in Table III should be discussed in greater depth. Furthermore, Supplementary Table 1 provides material for several additional analyses that were not explored in the manuscript.

d) References;

OK.

e) Figures and tables.

It is recommended that the authors verify the formatting of the tables, as some (e.g., Table IV) appear to be cropped in the version provided to the reviewers, limiting proper visualization.

## REVIEWER #2

The manuscript "Association of p-glycoprotein and bile salt export pump gene polymorphisms with advanced liver disease in hepatitis C virus infected patients" reports an observational retrospective study aimed at investigating the impact of *ABCB1* and *ABCB11* SNPs on disease progression in chronic HCV patients. Unfortunately, the manuscript presents important limitations in both study design and statistical analysis. Below, I list major comments that should be addressed before the manuscript can be considered for publication.

Study design: The description of the study design is insufficient. The primary outcome is not clearly defined, and the central hypothesis remains unclear. The recruitment of 232 HCV-infected participants and the extraction of clinical and demographic data from medical records are described, but the rationale for the comparisons performed is not adequately justified. The decision to compare *ABCB1* and *ABCB11* genotype frequencies across all extracted variables, including demographic factors such as sex and age, needs to be supported by a clear analytical framework.

2. Statistical analysis / Results:

Table III: HWE analysis must be performed using absolute genotype counts (observed and expected).

Table IV: Multivariate models should be applied to control for confounding variables. For example, cholesterol levels are influenced by factors such as age and sex. It should be made clear whether the reported associations remain significant after adjustment. Additionally, the table should be presented in landscape format, as some columns were hidden in the PDF version and could not be reviewed.

Table V represents a "combination of genotypes", and not a haplotype analysis. The authors compared individuals homozygous for all alternative alleles with those carrying at least one wild-type genotype. In fact, as *ABCB1* and *ABCB11* are located on different chromosomes, their variants cannot be combined in haplotypes.

## Editorial Decision and Recommendations

Thank you for submitting your manuscript "Association of p-glycoprotein and bile salt export pump gene polymorphisms with advanced liver disease in hepatitis C virus infected patients" to Memórias do Instituto Oswaldo Cruz. I have carefully evaluated the manuscript along with the reviewers' comments. While the study addresses an important topic in hepatology and genetics, several significant concerns must be addressed before the manuscript can be considered for publication.

Endorsement and Expansion of Reviewer Comments

I fully endorse the reviewers' assessments and would like to reinforce and expand on several critical points:

Study Design and Methodological Concerns

The reviewers correctly identified that the study design lacks sufficient clarity regarding the primary outcome and central hypothesis. The manuscript states it aims "to investigate the impact of *ABCB1* and *ABCB11* SNPs on disease progression in chronic HCV patients," but fails to:

Clearly define what constitutes "disease progression" as the primary endpoint

Justify the selection of specific variables for analysis among the many clinical parameters collected

Present a clear analytical framework that would explain why certain variables were prioritized over others

The retrospective nature of the study is acceptable, but the authors must explicitly state whether this was a hypothesis-driven investigation or an exploratory analysis. If exploratory, appropriate corrections for multiple comparisons must be applied and clearly documented.

Statistical Analysis Limitations

Reviewer 2 correctly identified several statistical issues that require attention:

Hardy-Weinberg Equilibrium analysis: Table III should present HWE analysis using absolute genotype counts (observed vs. expected), not percentages. The current presentation obscures the actual statistical test performed.

Lack of multivariate analysis: The univariate analyses presented in Tables IV and V cannot establish independent associations between the SNPs and clinical parameters. Given that factors such as age, sex, BMI, and diabetes status significantly influence lipid profiles and liver parameters, multivariate regression models controlling for these confounders are essential. For example, the association between 2677TT genotype and lower cholesterol levels may simply reflect age or diabetes differences between genotype groups rather than a direct genetic effect.

Multiple comparisons issue: With the large number of variables analyzed (28+ parameters across multiple SNPs), the risk of type I errors (false positives) is substantial. The authors must either apply appropriate corrections (e.g., Bonferroni, Benjamini-Hochberg) or explicitly acknowledge this limitation and frame their findings as hypothesis-generating rather than conclusive.

Misleading "haplotype" terminology: Reviewer 2 is absolutely correct that Table V does not represent a true haplotype analysis. Since *ABCB1* (chromosome 7) and *ABCB11* (chromosome 2) are located on different chromosomes, they segregate independently and cannot form haplotypes. The authors should reframe this as a "combined genotype analysis" or "genetic risk score" rather than using the technically incorrect term "haplotype."

Presentation and Interpretation Issues

Missing SNP in Table IV: As Reviewer 1 noted, the c.3435C>T polymorphism is discussed in the text but absent from Table IV without explanation. If no significant associations were found, this should be explicitly stated.

Clinical relevance considerations: The manuscript notes that "after the sample collection period, the patients received direct-acting antivirals (DAAs), and 99.3% achieved SVR." This important finding significantly impacts the interpretation of results, as it suggests these polymorphisms did not affect response to modern therapy. The authors should more thoroughly discuss the clinical relevance of their findings in the context of current DAA-based treatment paradigms.

Table formatting: Several tables (particularly Table IV) appear cropped in the current version, making proper evaluation difficult. All tables should be reformatted for clarity, with Table IV presented in landscape orientation as suggested.

## AUTHORS' RESPONSE TO THE REVIEWERS

**Manuscript ID:** MIOC-2025-0173

**Title:** Association of p-glycoprotein and bile salt export pump gene polymorphisms with advanced liver disease in hepatitis C virus infected patients

We would like to thank you for your valuable contributions, constructive suggestions and detailed comments that significantly enriched the quality of this work. We inform you that all requests and recommendations were met, and the main changes are highlighted in yellow in the manuscript.

**Reviewer #1**

Reviewer comments:

a) Adequacy of the abstract: Considering the elements highlighted in the abstract's structure, the content appears appropriate and accurately reflects the methodology and results described in the study.

*Response:* We appreciate the reviewer's positive assessment. We are pleased that the abstract was considered appropriate and consistent with the study's methodology and results.

b) Originality and importance of the contribution for the development of the field of study. And relevance of: Regarding originality, I note that the topic has been widely discussed in literature, particularly in relation to genetic polymorphisms in ABC genes and their impact on Hepatitis C severity. However, this study brings a relevant contribution by analyzing a Brazilian cohort and correlating the polymorphism data with clinical and laboratory findings. The results are thoroughly discussed, including the study's limitations. I consider this work relevant and of interest to the field.

*Response:* We thank the reviewer for recognizing the relevance and contribution of our study.

c) Methodology, results and discussion: In terms of methodology, the study presents a well-defined and clearly described approach. The results of the polymorphism analysis using the PCR technique are thoroughly explored, both in the narrative and in the accompanying figures. The discussion of the results is well developed, as it effectively correlates the data obtained from the different haplotypes evaluated in the study with the laboratory findings related to lipid profiles and hepatic parameters, including alpha fetoprotein as a biomarker for hepatocellular carcinoma. However, in the discussion of the results, it is not clear why the ABCB1 gene SNP -3435C>T was not included in Table IV. If no significant difference was observed, this should have been explicitly stated in the text. I also believe that the data presented in Table III should be discussed in greater depth. Furthermore, Supplementary Table 1 provides material for several additional analyses that were not explored in the manuscript.

*Response:* We thank the reviewer for the constructive feedback and positive comments on our methodology and discussion. The *ABCB1* 3435C>T was not included in Table IV because no statistically significant associations were identified. To improve clarity, we have now explicitly stated this information in the Results section, and we clarify in the table legend that Table IV presents only variables and SNPs with significant differences. In Results: "Table IV includes only the variables that showed statistically significant differences according to genotype or allele. In addition, only the SNPs that presented significant associations are displayed in this table, specifically *ABCB1* 2677G>T/A, *ABCB1* 1236C>T, and *ABCB11* 1331T>C." And in Table IV Legend: "No significant results were found for the analysis by allele for the c.1331T>C polymorphism; therefore, this SNP was not included in Table IV." In the revised manuscript, we also have expanded the discussion of the results presented in Table III (Hardy-Weinberg equilibrium). Discussion: "All variants were in Hardy–Weinberg equilibrium, indicating that the observed genotype frequencies were consistent with those expected for a genetically stable population. This supports the reliability of the genotyping procedures and suggests that no population stratification or methodological bias significantly affected the distribution of these SNPs, thereby reinforcing the validity of the association analyses performed." In the revised manuscript, we included in discussion more exploratory analysis with the data presented in supplementary table S1.

d) References: OK

*Response:* We appreciate the reviewer's positive evaluation regarding the references.

e) Figures and tables: It is recommended that the authors verify the formatting of the tables, as some (e.g., Table IV) appear to be cropped in the version provided to the reviewers, limiting proper visualization.

*Response:* We thank the reviewer for noting this issue. We have carefully reviewed and corrected the formatting of all tables in the revised version.

**Reviewer #2**

Reviewer comments:

The manuscript "Association of p-glycoprotein and bile salt export pump gene polymorphisms with advanced liver disease in hepatitis C virus infected patients" reports an observational retrospective study aimed at investigating the impact of *ABCB1* and *ABCB11* SNPs on disease progression in chronic HCV patients. Unfortunately, the manuscript presents important limitations in both study design and statistical analysis. Below, I list major comments that should be addressed before the manuscript can be considered for publication.

*Response:* We appreciate the constructive comments and have revised the manuscript accordingly to address the reviewer's concerns. In the following sections, we provide detailed responses and indicate the specific changes made to the text.

Study design: The description of the study design is insufficient. The primary outcome is not clearly defined, and the central hypothesis remains unclear. The recruitment of 232 HCV-infected participants and the extraction of clinical and demographic data from medical records are described, but the rationale for the comparisons performed is not adequately justified. The decision to compare *ABCB1* and *ABCB11* genotype frequencies across all extracted variables, including demographic factors such as sex and age, needs to be supported by a clear analytical framework.

*Response:* We thank the reviewers for their thorough evaluation and constructive comments regarding the study design. In the revised manuscript, we have clarified both the central hypothesis and the analytical framework guiding the study. First, we now explicitly define "disease progression" as our primary outcome, operationalized as the presence of advanced fibrosis (F3) or cirrhosis. Second, we have expanded the Methods section to clearly state that the study was hypothesis-driven, based on prior evidence suggesting that *ABCB1* and *ABCB11* polymorphisms may influence hepatic injury. Accordingly, we justified the selection of the clinical and laboratory variables analyzed as secondary outcomes, as these parameters (e.g., ALT, AST, GGT, AFP, lipid profile) are biologically linked to liver damage and metabolic alterations. We also justified the comparisons between genetic polymorphisms and demographic variables. This analysis was performed because these variables are well established confounders in liver disease progression and secondary outcomes. We revised the manuscript to present a more explicit analytical framework, explaining the rationale for prioritizing specific variables and how these relate to the functional consequences of the polymorphisms studied. Finally, to address the reviewer's concern about the number of statistical comparisons, we now acknowledge this limitation and describe the approach used.

2. Statistical analysis / Results: Table III: HWE analysis must be performed using absolute genotype counts (observed and expected).

*Response:* We thank the reviewer for this important observation. In the revised manuscript, Table III has been updated to present the Hardy–Weinberg Equilibrium (HWE) analysis using absolute genotype counts (both observed and expected), as recommended. The chi-square values and corresponding p-values have also been included to ensure full transparency of the statistical test performed.

Table IV: Multivariate models should be applied to control for confounding variables. For example, cholesterol levels are influenced by factors such as age and sex. It should be made clear whether the reported associations remain significant after adjustment. Additionally, the table should be presented in landscape format, as some columns were hidden in the PDF version and could not be reviewed.

*Response:* We thank the reviewer for this very important observation and suggestion. In the revised manuscript, we performed a multivariable analysis that included the relevant confounding variables. The methodology used for this analysis is specified in the Methods section. The results of the multivariate analysis are described in the Results section, under Statistical Analysis, and are presented in Tables IV and V. Finally, we reformatted Table IV in landscape orientation to prevent loss of information and improve readability.

Table V represents a "combination of genotypes", and not a haplotype analysis. The authors compared individuals homozygous for all alternative alleles with those carrying at least one wild-type genotype. In fact, as *ABCB1* and *ABCB11* are located on different chromosomes, their variants cannot be combined in haplotypes.

*Response:* We thank the reviewer for this important clarification. We agree that Table V does not represent a true haplotype analysis, since *ABCB1* (chromosome 7) and *ABCB11* (chromosome 2) segregate independently. Accordingly, and following the reviewer's suggestion, we have replaced the term "haplotype analysis" with "combined genotype analysis" throughout the manuscript, including in the Abstract, Results, Discussion, and in Table V.

# Reviewer report: REVIEWERS' COMMENTS

## REVIEWER #1

Dear Editor, upon careful consideration of the comments provided by Reviewer 2, I identified several aspects that had not been fully addressed in my initial assessment. These points of revision clearly contribute to improving data transparency and strengthening the overall robustness of the manuscript. In conclusion, after addressing all comments, the authors have incorporated the suggested modifications, and I consider the manuscript suitable for publication.

## REVIEWER #2

The manuscript entitled "Association of p-glycoprotein and bile salt export pump gene polymorphisms with advanced liver disease in hepatitis C virus infected patients" is relevant, since genetic association analyses with the presented outcome (and other related phenotypes) are still scarce for the Brazilian population.

After reviewing the latest version, I agreed with all the reviewers' and editor's comments. However, I will add some suggestions and have some questions that I will point out below.

Review all gene names (abbreviations): italicized.

The word mutation/mutated is no longer commonly used when discussing association studies. The most commonly used term is variant or genetic variant (including for monogenic traits), although the term polymorphism is still used. I suggest reviewing these terms throughout the text, tables, and figures.

Check where the term "population" appears. "Sample" or "cohort" would be better.

The main point that needs to be reviewed relates to the statistical analysis of association. Depending on how the analyses were performed, there will be changes in the writing of the abstract, results and discussion, as well as the tables.

In Table IV, it was not clear how the means/standard deviations were obtained. Were heterozygotes counted in both groups? I suggest performing an analysis using dominant and recessive models, to always have a group as a reference and not repeat heterozygotes in the analyses.

In the analysis of combined genotypes, it was not clear in the table how this combination of genotypes occurred. For example, see Discussion: "The first group comprised individuals with a 100% mutant genotype (TTTC); that is, those who carried the mutant genotype of all four SNPs (1236TT, 2677TT, 3435TT and 1331CC). The second group comprised patients with other possible combinations of genotypes; that is, individuals who had the wild-type genotype for at least one of the SNPs studied." If this is what was done, N should be at most 21 (due to the lower frequency of the TT genotype for G2677T). Table V (n=88 for 100% mutant genotype) suggests that an analysis was performed regarding the presence of at least one mutant "allele" (TTTC). In this case, how were the averages calculated for the two groups, given that we are talking about the number of "alleles" (n=88 and 376) and not the number of individuals?

Table IV: For the polymorphism c.1331T>C, the numbers (N) are wrong: N=270 is in relation to the 1331C allele and N=194 is in relation to the 1331T allele.

Legend: "No significant results were found for the analysis by allele for c.1331T>C..." Shouldn't the SNP be c.3435C>T? On the third line: "The result presented is for comparison with the analysis by genotype." This sentence should be deleted because allele analyses were also performed.

## AUTHORS' RESPONSE TO THE REVIEWERS

Name of Journal: Memórias do Instituto Oswaldo Cruz Manuscript ID: MIOC-2025-0173 Title: Association of p-glycoprotein and bile salt export pump gene polymorphisms with advanced liver disease in hepatitis C virus infected patients We would like to thank the reviewer for the valuable contributions, constructive suggestions, and careful evaluation of the manuscript, which significantly improved its clarity, transparency, and overall quality. We are pleased to inform you that all comments and recommendations have been fully addressed in the revised version of the manuscript, and the main changes are highlighted (Text Highlight Color) in yellow.

**Reviewer #1**

Reviewer comment: Dear Editor, upon careful consideration of the comments provided by Reviewer 2, I identified several aspects that had not been fully addressed in my initial assessment. These points of revision clearly contribute to improving data transparency and strengthening the overall robustness of the manuscript. In conclusion, after addressing all comments, the authors have incorporated the suggested modifications, and I consider the manuscript suitable for publication.

*Response:* We thank the reviewer for the careful re-evaluation of our manuscript and for considering the additional points raised by Reviewer 2. We appreciate the recognition that addressing these comments contributed to improving data transparency and strengthening the overall robustness of the study. We are grateful for the positive assessment and for considering the revised manuscript suitable for publication after the incorporation of the suggested modifications.

**Reviewer #2**

Reviewer comments: The manuscript entitled "Association of p-glycoprotein and bile salt export pump gene polymorphisms with advanced liver disease in hepatitis C virus infected patients" is relevant, since genetic association analyses with the presented outcome (and other related phenotypes) are still scarce for the Brazilian population. After reviewing the latest version, I agreed with all the reviewers' and editor's comments. However, I will add some suggestions and have some questions that I will point out below.

a) Review all gene names (abbreviations): italicized.

*Response:* We thank the reviewer for the careful observation regarding gene nomenclature. All gene names (*ABCB1* and *ABCB11*) were reviewed and are now consistently formatted in italics throughout the manuscript, following international standards. All suggested alterations were incorporated throughout the manuscript.

b) The word mutation/mutated is no longer commonly used when discussing association studies. The most commonly used term is variant or genetic variant (including for monogenic traits), although the term polymorphism is still used. I suggest reviewing these terms throughout the text, tables, and figures.

*Response:* Throughout the manuscript, the terms "mutation" and "mutant" were replaced by "variant", "variant genotype" or polymorphism, in accordance with current recommendations for genetic association studies. All suggested alterations were incorporated throughout the manuscript.

c) Check where the term "population" appears. "Sample" or "cohort" would be better.

*Response:* We thank the reviewer for this important suggestion. As recommended, the terminology "population" was carefully revised and replaced throughout the manuscript with "study sample" or "cohort," as appropriate, to better reflect the nature of our study. All suggested alterations were incorporated throughout the manuscript.

d) The main point that needs to be reviewed relates to the statistical analysis of association. Depending on how the analyses were performed, there will be changes in the writing of the abstract, results and discussion, as well as the tables.

*Response:* The manuscript was carefully revised to standardize and clarify the terminology used to describe genetic association analyses. The text now consistently distinguishes between genotype- and allele-based analyses and avoids ambiguous terminology. Corresponding revisions were implemented throughout the Abstract, Results, Discussion, and tables to ensure full coherence between the statistical analyses performed and their interpretation. All suggested alterations were incorporated throughout the manuscript.

e) In Table IV, it was not clear how the means/standard deviations were obtained. Were heterozygotes counted in both groups? I suggest performing an analysis using dominant and recessive models, to always have a group as a reference and not repeat heterozygotes in the analyses.

*Response:* We thank the reviewer for this important comment regarding the calculation of means and standard deviations and the analytical approach used in article. In the genotype-based analysis, each patient was considered as a single observational unit and categorized according to their genotype. The wild-type genotype was used as the reference group, and comparisons were performed between wild-type versus heterozygous and wild-type versus mutant genotypes. In this approach, means and standard deviations were calculated per individual, and heterozygotes were not counted more than once. In the allele-based analysis, the unit of analysis was the allele rather than the individual. Accordingly, the dataset was expanded so that each individual contributed two alleles, allowing comparisons between wild-type and variant alleles. In this case, means and standard deviations were calculated based on allele counts, and not on the number of patients. We have revised the Methods section and the table legends to explicitly describe these analytical strategies and clarify how summary statistics were obtained, thereby ensuring transparency and avoiding misinterpretation.

f) In the analysis of combined genotypes, it was not clear in the table how this combination of genotypes occurred.

*Response:* We thank the reviewer for this important observation. We acknowledge that the terminology used in the previous version was inaccurate. The analysis presented does not correspond to a combined genotype (individual-based) analysis, but rather to a combined allele analysis. In this analysis, the unit of observation was the allele, not the individual. Accordingly, the groups were defined as follows: Group 1: alleles carrying the variant form for all four polymorphisms studied (1236T and 2677T and 3435T and 1331C); Group 2: alleles presenting at least one wild-type allele among the four loci (1236C or 2677G or 3435C or 1331T). We have corrected the terminology throughout the manuscript, tables, and figure legends, replacing "combined genotype analysis" with "combined allele analysis", and we have clarified the definition of the comparison groups to avoid further misunderstanding.

g) ABSTRACT: The sentences "Most frequent *ABCB1* SNP was c.3435C>T (TT; 15.5%), followed by c.1236C>T (TT; 11.2%) and c.2677G>T (TT; 9.0%). *ABCB11* c.1331T>C was found in 31.3%." should be revised.

*Response:* We thank the reviewer for the suggestion. The Abstract was revised accordingly, and the frequencies were corrected to report variant alleles as recommended: "The most frequent *ABCB1* variant allele was c.3435C>T (36.8%), followed by c.1236C>T (30.8%) and c.2677G>T (26.7%). The *ABCB11* c.1331CC genotype was observed in 31.3% of the cohort."

h) Sponsorships: "Concelho": CONSELHO.

*Response:* We thank the reviewer for pointing out this typographical error. The word 'Concelho' has been corrected to 'CONSELHO' in the revised manuscript.

i) INTRODUCTION: In some locations c.2677G>T, in others c.2677G>T/A.

*Response:* We thank the reviewer for pointing out this inconsistency. The entire manuscript was carefully reviewed, and the nomenclature was standardized to c.2677G>T throughout. All suggested alterations were incorporated throughout the manuscript.

j) MATERIAL AND METHODS: Suggestion: "Extraction and analysis of the *ABCB1* and *ABCB11* genes" should be changed to "DNA Extraction and analysis of the *ABCB1* and *ABCB11* genes". Wouldn't the kit used for extraction be the QIAamp DNA Mini Kit, and not the QIAamp RNA Mini Kit?

*Response:* We thank the reviewer for this important observation. The extraction kit used in this study does not separate genetic material by type (RNA versus DNA); therefore, it allows the simultaneous extraction of both nucleic acids from the same biological sample. As hepatitis C virus is an RNA virus and constitutes the basis of this study, the QIAamp RNA Mini Kit was intentionally used, as it enables downstream analyses of viral RNA as well as host genomic DNA from the same extracted material. According to the manufacturer (QIAGEN), this kit is suitable for the extraction of both RNA and DNA. In addition, we have revised the wording as suggested, and the section now reads "Nucleic Acid extraction and analysis of the *ABCB1* and *ABCB11* genes" in the revised manuscript.

k) Suggestion: "genes was performed using real-time polymerase chain reaction with the TaqMan Universal PCR Master Reagent Mix (Applied Biosystems, Foster City, CA, United States) and the TaqMan SNP Genotyping Assay kit (Applied Biosystems) for allele discrimination for each polymorphism, according to the manufacturer's instructions."

*Response:* We thank the reviewer for the suggestion. The wording was revised as recommended.

l) Table 1 is not necessary and could be a supplementary table, with the assay number for each polymorphism.

*Response:* We thank the reviewer for this valuable suggestion. The manuscript was revised accordingly: Table 1 was removed from the main text and included as Supplementary Table S1.

m) RESULTS: Suggestion: "Statistical analysis" for "Association analysis". Page 10: Line 29: "Furthermore, significantly lower levels of HDL (P < 0.05) were associated with the 2677TT mutation and 2677T allele." To: "Furthermore, significantly lower levels of HDL (P < 0.05) were associated with the 2677TT GENOTYPE and 2677T allele."

*Response:* We thank the reviewer for the suggestion. The section title was revised from "Statistical analysis" to "Association analysis," and the wording was corrected to replace "mutation" with "genotype," as recommended.

t) Table II: Remove "n (%)" from the title. Not all variables are represented by "n (%)", some, for example albumin, are represented by the mean +/- standard deviation. Therefore, the second column of the table should not have the title "Frequency".

*Response:* We thank the reviewer for this suggestion. The term "Frequency" was replaced with "Value." The table legend was revised to clarify that data are presented as n (%) or mean ± standard deviation, as appropriate.

u) Table III: Not necessary in the manuscript. Normally in this type of study, only Hardy-Weinberg equilibrium is mentioned in the text.

*Response:* We thank the reviewer for this comment. To improve transparency, Table III was not removed but relocated to the Supplementary Material (Table S3). The main results regarding Hardy–Weinberg equilibrium were maintained and clearly described in the main text.

v) Table IV: For the polymorphism c.1331T>C, the numbers (N) are wrong: N=270 is in relation to the 1331C allele and N=194 is in relation to the 1331T allele. Legend: "No significant results were found for the analysis by allele for c.1331T>C..." Shouldn't the SNP be c.3435C>T?

*Response:* We thank the reviewer for these important observations. The allele frequencies for the c.1331T>C polymorphism, which were previously reversed, have been corrected in Table IV. In addition, the suggested changes to the legend were implemented: the SNP notation was revised, and the sentence referring to comparison with genotype analysis was removed.

# Reviewer report: EDITORIAL DECISION AND RECOMMENDATIONS

## Editorial Decision and Recommendations

Dear Authors,

Thank you for the opportunity to review the revised version of your manuscript (MIOC-2025-0173.R2). I would like to commend you for the substantial effort put into addressing the previous comments. The clarification of the study design—specifically the explicit definition of the primary outcome (advanced fibrosis/cirrhosis) versus secondary outcomes—and the distinction between hypothesis-driven and exploratory components have significantly strengthened the manuscript's methodological framework. Furthermore, the correction of the "haplotype" terminology to "combined allele analysis" and the expanded discussion on the clinical relevance in the context of DAA therapy are excellent improvements that enhance the scientific accuracy and impact of the study.

However, upon careful comparison of the revised text with the specific concerns raised in the First Round of Review, there are two critical points that remain partially unaddressed. To ensure the manuscript meets the high standards required for publication, I request that you address the following:

1. Explicit Statement Regarding the c.3435C>T Polymorphism Reviewer 1 (Round 1) Comment c) noted that it was unclear why the *ABCB1* gene SNP -3435C>T was not included in Table IV. The reviewer specifically requested that if no significant difference was observed, this should be explicitly stated in the text.

Current Status: In the revised Results and Abstract, you report associations for c.1236C>T, c.2677G>T, and c.1331T>C, as well as the combined allele analysis. However, the c.3435C>T polymorphism is conspicuously absent from the summary of findings without an explanation.

Recommendation: Please add a sentence in the Results section and/or a footnote in the table explicitly addressing this SNP.

Suggested addition (Results): "Regarding the *ABCB1* c.3435C>T polymorphism, no statistically significant associations were observed with any of the clinical or laboratory parameters evaluated (data not shown)."

2. Presentation of Multivariate Analysis Results Reviewer 2 (Round 1) and the Editorial Decision strongly emphasized the necessity of multivariate regression models to control for confounding variables (age, sex, BMI, diabetes) and requested that the results be presented to show if associations remain significant after adjustment.

Recommendation: To fully satisfy the reviewer's concern regarding confounding factors, you must report the results of the multivariate analysis for the significant findings. Table IV: Please update Table IV to include columns for the multivariate analysis (e.g., adjusted Odds Ratios, Beta coefficients, and P-values) alongside the descriptive statistics. Alternatively, if Table IV is intended for descriptive data only, create a new table for the adjusted associations. Text Revision: Ensure the Results text specifies whether the associations held up after adjustment.

Suggested revision (Results): "Genotypes 1236TT and 2677TT and their alleles were associated with lower total cholesterol even after adjusting for age, sex, BMI, and diabetes status (P < 0.05)." (If the adjustment changed the significance, this must also be stated).

By addressing these two points, you will provide the transparency regarding negative findings (3435C>T) and the statistical robustness (multivariate adjustment) that were requested in the initial review.

Best regards,

Dr. Marcelo Ribeiro Alves

Handling Editor

Memórias do Instituto Oswaldo Cruz

## AUTHORS' RESPONSE TO THE REVIEWERS

Name of Journal: Memórias do Instituto Oswaldo Cruz Manuscript ID: MIOC-2025-0173 Title: Association of p-glycoprotein and bile salt export pump gene polymorphisms with advanced liver disease in hepatitis C virus infected patients

Dear Dr. Marcelo Ribeiro Alves,

We sincerely thank you for the careful handling of our manuscript and for coordinating the review process. We are also grateful to the reviewers for their valuable contributions, constructive suggestions, and thorough evaluation, which significantly improved the clarity, transparency, and overall quality of our work. We are pleased to inform you that all comments and recommendations have been carefully addressed in the revised version of the manuscript.

Comments: Dear Authors, Thank you for the opportunity to review the revised version of your manuscript (MIOC-2025-0173.R2). I would like to commend you for the substantial effort put into addressing the previous comments. The clarification of the study design—specifically the explicit definition of the primary outcome (advanced fibrosis/cirrhosis) versus secondary outcomes—and the distinction between hypothesis-driven and exploratory components have significantly strengthened the manuscript's methodological framework. Furthermore, the correction of the "haplotype" terminology to "combined allele analysis" and the expanded discussion on the clinical relevance in the context of DAA therapy are excellent improvements that enhance the scientific accuracy and impact of the study. However, upon careful comparison of the revised text with the specific concerns raised in the First Round of Review, there are two critical points that remain partially unaddressed. To ensure the manuscript meets the high standards required for publication, I request that you address the following:

1. Explicit Statement Regarding the c.3435C>T Polymorphism Reviewer 1 (Round 1) Comment c) noted that it was unclear why the *ABCB1* gene SNP -3435C>T was not included in Table IV. The reviewer specifically requested that if no significant difference was observed, this should be explicitly stated in the text.

*Response:* We thank you for highlighting the importance of ensuring full transparency regarding the *ABCB1* c.3435C>T polymorphism. We would like to clarify that, in the previous revision, we had already specified that this SNP was not included in the main table because no statistically significant associations were observed. This information was provided both in the Results section and in the legend of Table II. To further enhance clarity and avoid any possible ambiguity, we have now added the following explicit sentence to the Results section: "Regarding the *ABCB1* c.3435C>T polymorphism, no statistically significant associations were observed with any of the clinical or laboratory parameters evaluated (data not shown)." We believe this addition ensures complete transparency regarding the negative findings and fully addresses the concern raised.

2. Presentation of Multivariate Analysis Results Reviewer 2 (Round 1) and the Editorial Decision strongly emphasized the necessity of multivariate regression models to control for confounding variables (age, sex, BMI, diabetes) and requested that the results be presented to show if associations remain significant after adjustment.

*Response:* We thank you for emphasizing the importance of clearly presenting the multivariate regression results. We would like to clarify that, in the previous revision, a column containing the P values from the multivariate analyses had already been added to Tables II and III (formerly Tables IV and V). These adjusted P values correspond to multivariate models controlling for age, sex, body mass index (BMI), and diabetes status. In addition, the legends of both tables already included the statement: "In the multivariate analysis, potential confounders (age, sex, body mass index, and diabetes) were incorporated into the model to control for their effects." To further enhance clarity and ensure complete transparency regarding the adjusted analyses, we have now complemented the tables by adding the corresponding beta coefficients (β) and 95% confidence intervals to Tables II and III. These measures were also explicitly incorporated into the Results section, alongside the adjusted P values that were already reported. We believe these additions provide a more comprehensive presentation of the magnitude and direction of the associations and fully address the concern regarding multivariate adjustment and control of potential confounding factors.

## Handling Editor's comments

Dear Dr. Barros:

Thank you for your thoughtful and comprehensive responses to the reviewers' comments and for the careful revisions incorporated into the manuscript (MIOC-2025-0173.R3). I appreciate the transparency added regarding the *ABCB1* c.3435C>T polymorphism and the enhanced presentation of multivariate analyses. These modifications substantially improve the clarity and interpretability of your findings.

I am pleased to inform you that the manuscript is accepted for publication in Memorias do Instituto Oswaldo Cruz, in the certainty that the specific details regarding the presentation of statistical results pointed out below will be carefully addressed by the authors. These adjustments are essential to ensure the accuracy and clarity of the findings presented.

METHODOLOGICAL CONSIDERATION ON STATISTICAL REPORTING

Upon detailed review of the revised statistical reporting, I noted a methodological consideration regarding the presentation of results for binary outcomes. Specifically, for the analysis of cirrhosis (a dichotomous variable) in Table II, logistic regression was appropriately employed; however, the results are currently reported as beta coefficients with 95% CIs. For logistic regression models, the conventional and more interpretable measure of association is the Odds Ratio (OR) with its corresponding 95% CI, as this expresses the relative effect size compared to the reference category in a clinically meaningful manner.

Explanation for conversion: In logistic regression, beta represents the log-odds coefficient. The odds ratio is obtained by exponentiating beta: OR = exp(beta). Similarly, the 95% CI for the OR is calculated by exponentiating the lower and upper bounds of the beta coefficient's CI.

INCONSISTENCIES FOUND

I also observed inconsistencies in the reported values for the *ABCB11* c.1331T>C polymorphism and cirrhosis (genotype-based analysis). You reported "beta = -1.12; 95%CI: 0.14 to 2.14".

1. Confidence Interval: The reported CI (0.14 to 2.14) does not contain the point estimate beta = -1.12, suggesting a possible typographical error in the CI bounds.

2. Direction of Effect: A negative beta (-1.12) indicates a protective effect (OR < 1). However, your text states that the "variant genotype showing a higher frequency of cirrhosis," which implies a risk factor (OR > 1). This suggests a potential error in either the sign of the coefficient or the reference category used in your model.

Please revisit your statistical output to confirm the correct beta estimates and confidence intervals before applying the exponentiation transformation.

CALCULATIONS AND SUGGESTED REVISIONS

1. Results section text – Genotype-based analysis for Cirrhosis:

Assuming the intended CI for beta was approximately -2.14 to -0.14 (symmetric around -1.12), the conversion would be: OR = exp(-1.12) approx. 0.33; 95% CI_OR = [exp(-2.14), exp(-0.14)] approx. [0.12, 0.87]

Replace: "the association between the c.1331T>C variant and cirrhosis remained significant in the genotype-based analysis (CC: beta = -1.12; 95%CI: 0.14 to 2.14; p<0.05), with the variant genotype showing a higher frequency of cirrhosis." with "the association between the c.1331T>C variant and cirrhosis remained significant in the genotype-based analysis (CC: adjusted OR = 0.33; 95%CI: 0.12–0.87; p<0.05), with the variant genotype showing a higher frequency of cirrhosis."

(Note: Please verify if the OR is indeed < 1, as this implies protection. If the variant is a risk factor, the beta should be positive.)

2. Table II – *ABCB11* c.1331T>C SNP, cirrhosis, genotype-based analysis: Replace the phrase "< 0.05 (beta = -1.12; 95%CI: 0.14 to 2.14)" with "< 0.05 (adjusted OR = 0.33; 95%CI: 0.12–0.87)".

3. Table II – *ABCB11* c.1331T>C SNP, cirrhosis, allele-based analysis: Current reporting: 0.1 (beta = 0.41; 95%CI: -0.02 to 0.85). OR = exp(0.41) approx. 1.51; 95% CI_OR = [exp(-0.02), exp(0.85)] approx. [0.98, 2.34]. Replace: "0.1 (beta = 0.41; 95%CI: -0.02 to 0.85)" with "0.1 (adjusted OR = 1.51; 95%CI: 0.98–2.34)".

IMPORTANT CLARIFICATION

For continuous outcomes (e.g., total cholesterol, HDL, AST), the presentation of beta coefficients with 95% CIs from linear regression models is statistically appropriate and requires no modification. Specifically, the result for AST levels reported in the text as "beta = 9.44; 95%CI: 0.73 to 18.2" refers to a continuous outcome and is correct as stated.

These adjustments will align your manuscript with standard reporting guidelines for regression analyses in clinical research and ensure the highest quality for your publication.

Sincerely,

Dr. Marcelo Ribeiro Alves

Handling Editor

Memórias do Instituto Oswaldo Cruz
